# Prediction of incident cardiovascular events using machine learning and CMR radiomics

**DOI:** 10.1007/s00330-022-09323-z

**Published:** 2022-12-13

**Authors:** Esmeralda Ruiz Pujadas, Zahra Raisi-Estabragh, Liliana Szabo, Celeste McCracken, Cristian Izquierdo Morcillo, Víctor M. Campello, Carlos Martín-Isla, Angelica M. Atehortua, Hajnalka Vago, Bela Merkely, Pal Maurovich-Horvat, Nicholas C. Harvey, Stefan Neubauer, Steffen E. Petersen, Karim Lekadir

**Affiliations:** 1 Artificial Intelligence in Medicine Lab (BCN-AIM), Departament de Matemàtiques i Informàtica, Universitat de Barcelona, Barcelona, Spain; 2 William Harvey Research Institute, NIHR Barts Biomedical Research Centre, Queen Mary University of London, Charterhouse Square, London, EC1M 6BQ UK; 3 Barts Heart Centre, St Bartholomew’s Hospital, Barts Health NHS Trust, West Smithfield, London, EC1A 7BE UK; 4 Semmelweis University Heart and Vascular Center, Budapest, Hungary; 5 Division of Cardiovascular Medicine, Radcliffe Department of Medicine, University of Oxford, National Institute for Health Research Oxford Biomedical Research Centre, Oxford University Hospitals NHS Foundation Trust, Oxford, OX3 9DU UK; 6 Semmelweis University Medical Imaging Centre, Budapest, Hungary; 7 MRC Lifecourse Epidemiology Centre, University of Southampton, Southampton, UK; 8 NIHR Southampton Biomedical Research Centre, University of Southampton and University Hospital Southampton NHS Foundation Trust, Southampton, UK; 9 Health Data Research UK, London, UK; 10 Alan Turing Institute, London, UK

**Keywords:** Machine learning, Atrial fibrillation, Preventive medicine, Heart failure, Radiomics

## Abstract

**Objectives:**

Evaluation of the feasibility of using cardiovascular magnetic resonance (CMR) radiomics in the prediction of incident atrial fibrillation (AF), heart failure (HF), myocardial infarction (MI), and stroke using machine learning techniques.

**Methods:**

We identified participants from the UK Biobank who experienced incident AF, HF, MI, or stroke during the continuous longitudinal follow-up. The CMR indices and the vascular risk factors (VRFs) as well as the CMR images were obtained for each participant. Three-segmented regions of interest (ROIs) were computed: right ventricle cavity, left ventricle (LV) cavity, and LV myocardium in end-systole and end-diastole phases. Radiomics features were extracted from the 3D volumes of the ROIs. Seven integrative models were built for each incident cardiovascular disease (CVD) as an outcome. Each model was built with VRF, CMR indices, and radiomics features and a combination of them. Support vector machine was used for classification. To assess the model performance, the accuracy, sensitivity, specificity, and AUC were reported.

**Results:**

AF prediction model using the VRF+CMR+Rad model (accuracy: 0.71, AUC 0.76) obtained the best result. However, the AUC was similar to the VRF+Rad model. HF showed the most significant improvement with the inclusion of CMR metrics (VRF+CMR+Rad: 0.79, AUC 0.84). Moreover, adding only the radiomics features to the VRF reached an almost similarly good performance (VRF+Rad: accuracy 0.77, AUC 0.83). Prediction models looking into incident MI and stroke reached slightly smaller improvement.

**Conclusions:**

Radiomics features may provide incremental predictive value over VRF and CMR indices in the prediction of incident CVDs.

**Key Points:**

• *Prediction of incident atrial fibrillation, heart failure, stroke, and myocardial infarction using machine learning techniques*.

• *CMR radiomics, vascular risk factors, and standard CMR indices will be considered in the machine learning models*.

• *The experiments show that radiomics features can provide incremental predictive value over VRF and CMR indices in the prediction of incident cardiovascular diseases*.

**Supplementary Information:**

The online version contains supplementary material available at 10.1007/s00330-022-09323-z.

## Introduction

Cardiovascular disease (CVD) is the most common cause of morbidity and mortality worldwide [1]. Accurate risk stratification has a key role in ensuring appropriately targeted preventive strategies. Existing disease prediction algorithms reliant on demographic and clinical variables have been proposed for prediction of selected major CVDs [2–4].

Cardiovascular magnetic resonance (CMR) is the reference modality for quantification of cardiovascular structure and function and is widely used in clinical and research settings [5]. The rich phenotyping provided by CMR allows characterisation of pre-clinical organ-level remodelling [6]. Therefore, there is growing interest in the integration of imaging biomarkers into CVD prediction algorithms [7]. However, existing approaches to CMR image analysis are limited to simplistic volumetric measurements or qualitative assessments [8]. These conventional CMR metrics (left ventricular ejection fraction or maximal end-diastolic wall thickness) have shown potential for the early detection of cardiac deterioration and the characterisation of subclinical diseases [9].

Radiomics is a quantitative image analysis method, which allows extraction of highly detailed information about ventricular shape and myocardial character, thereby providing new information from existing standard-of-care images [10]. Radiomics features may be used as predictor variables in clinical models, often developed using machine learning (ML) methods. A key advantage of radiomics analysis over unsupervised ML algorithms is the interpretability of the models; that is, the radiomics features can be traced back to the heart’s morphological and tissue level alterations [11]. CMR radiomics is in the early stages of its development and thus far existing work has largely focused on demonstrating feasibility of the technique for disease discrimination [12, 13]. The CMR radiomics analysis is more mature within oncology and in this context, radiomics models have been successful for prediction of incident health events [14]. The value of CMR radiomics models for incident CVD prediction has not been previously studied.

In this work, we aim to evaluate the feasibility and clinical utility of CMR radiomics for the prediction of four key incident CVDs: atrial fibrillation (AF), heart failure (HF), myocardial infarction (MI), stroke. To evaluate the incremental value of CMR radiomics over existing approaches, we hierarchically built supervised ML models incorporating traditional vascular risk factors (VRFs) and conventional CMR metrics.

## Methods

### Population and setting

The UK Biobank (UKB) is an extensive cohort study that comprises over half a million individuals recruited between 2006 and 2010. The UKB provides a rich source of health data including comprehensive medical history, risk factors, biomarkers, and physical measurements [15]. The UKB imaging study commenced in 2015 and aims to scan 100,000 participants from the original dataset, and includes CMR [16]. Participants’ incident outcomes are tracked through the national data sources, including Hospital Episode Statistics (HES) and death registers to provide continuous longitudinal follow-up [17].

### Ethical approval

This study complies with the Declaration of Helsinki; the work was covered by the ethical approval for UKB studies from the National Health Service (NHS) National Research Ethics Service on 17 June 2011 (Ref 11/NW/0382) and extended on 18 June 2021 (Ref 21/NW/0157) with written informed consent obtained from all participants.

### Definition of the study sample

**Fig. 1:**
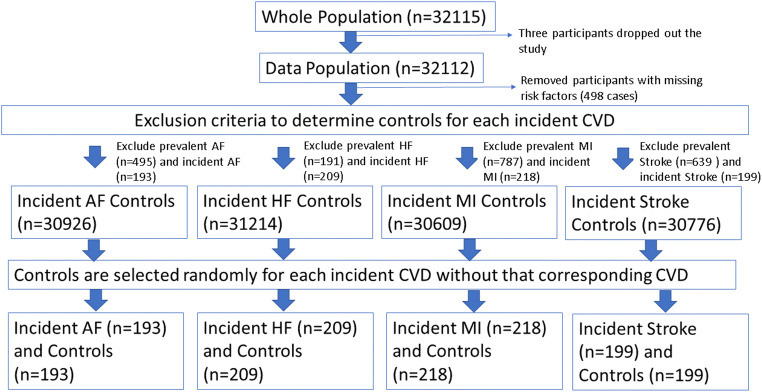
Definition of the study sample. Abbreviations: AF, atrial fibrillation; HF, heart failure; MI, myocardial infarction

From the UK Biobank, most of the participants start with a healthy condition developing diseases along the time. We identified individuals who experienced incident AF (*N* = 193), HF (*N* = 209), MI (*N* = 218), or stroke (*N* = 199) until the censoring date, 28 February 2021. Outcomes were ascertained through linked HES data with diseases defined according to the standardised International Classification of Diseases (ICD) codes (Supplementary Table 1). Individuals with the outcome of interest at imaging were not included. We selected comparator groups for each outcome (AF, HF, MI, stroke) comprising an equal number of randomly selected subjects who did not develop the outcome of interest during follow-up to eliminate class imbalance bias (Fig. 1).

### Vascular risk factors

We selected VRFs based on biological plausibility and reported associations in the literature, including the following variables: age, sex, body mass index, material deprivation, education, current smoking, alcohol intake, physical exercise, high cholesterol, diabetes mellitus, and hypertension [18]. The definition used for the ascertainment of high cholesterol, diabetes mellitus, and hypertension is given in Supplementary Table 1.

### Conventional CMR measures

All CMR scans were completed in dedicated UKB imaging centres using 1.5-T scanners (MAGNETOM Aera, Syngo Platform VD13A, Siemens Healthcare) under pre-defined acquisition protocols [19]. Standard long-axis images and a short-axis stack covering both ventricles from base to apex were captured using balanced steady-state free precession sequence [19]. CMR examinations of the first 5065 UKB participants were assessed manually using CVI42 post-processing software (version 5.1.1, Circle Cardiovascular Imaging Inc.) [20]. This analysis set was used to develop a fully automated quality-controlled pipeline and extract the contours for the 32,121 CMR studies [21, 22].

The following conventional CMR indices were considered during our analysis: LV end-diastolic volume (LVEDV), LV end-systolic volume (LVESV), RV end-diastolic volume (RVEDV), RV end-systolic volume (RVESV), LV stroke volume (LVSV), RV stroke volume (RVSV), LV ejection fraction (LVEF), RV ejection fraction (RVEF), LV mass (LVM). For ease of interpretation, we gave LV and RV ventricular volumes and masses in body surface area standardised format.

### Background of CMR radiomics

CMR radiomics is a novel image analysis technique permitting the computation of multiple indices of shape and texture [10]. Three classes of features are extracted: shape, first-order, and texture-based features. First-order features are histogram-based and related to the distribution of the grey level values in the tissue. Shape features describe geometrical properties of the organ, such as volume, diameter, minor/major axis, and sphericity. Texture features are derived from images that encode the global texture information, using mathematical formulae based on the spatial arrangement of pixels. Radiomics features can appreciate the heart’s complexity in detail by revealing patterns invisible to the naked eye. Thus, it furnishes a nearly limitless supply of imaging biomarkers with potential added value over conventional CMR metrics. However, caution should be taken regarding the reproducibility of different features [23].

### Radiomics feature extraction

**Fig. 2:**
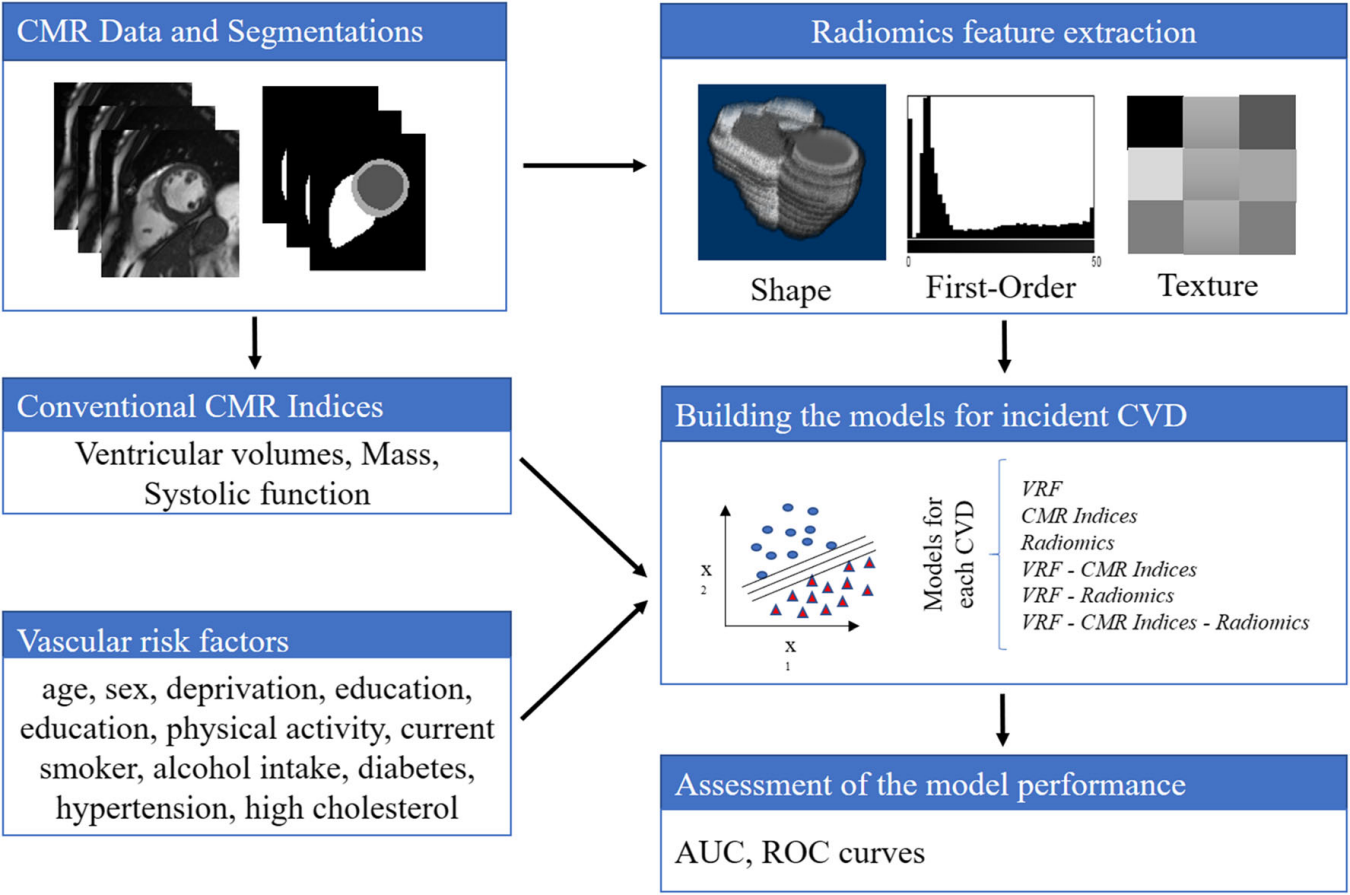
Flowchart to create the models for incident CVD. Abbreviations: CMR, cardiac magnetic resonance imaging; CVD, cardiovascular disease; VRF, vascular risk factor

The radiomics workflow is illustrated in Fig. 2. We used the short-axis stack contours for conventional image analysis to define three regions of interest (ROIs) for radiomics analysis: RV cavity, LV cavity, LV myocardium in ES and ED phases. We calculated these features from the 3D volumes of the ROIs. The open-source PyRadiomics platform (version 2.2.0.) was adopted to extract radiomics features. The grey value discretisation was performed using a binwidth of 25 to pull the intensity-based and texture radiomics features. A total of 262 radiomics features were included from each CMR study (LV shape *n* = 26, RV shape *n* = 26, MYO shape *n* = 26, LV myocardium first-order *n* = 36, LV myocardium texture *n* = 148).

### Radiomics feature selection

**Fig. 3:**
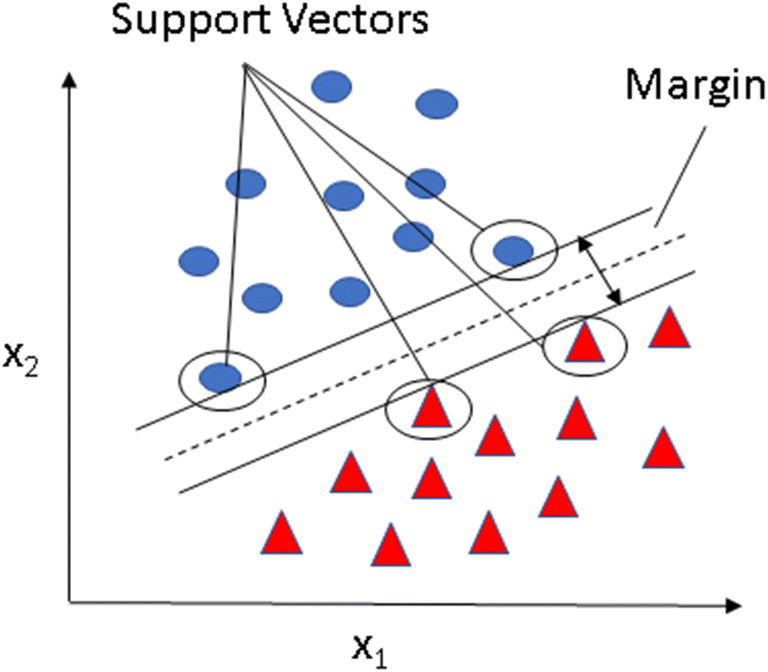
SVM process of maximising the margin. The objective of the support vector machine model is to maximise the margin between cases and controls, which is defined as the distance between the separating hyperplane (decision boundary) and the training samples that are closest to this hyperplane, which is the so-called support vectors (marked with circles)

Sequential feature forward selection (SFFS) algorithm [24] was applied to select the most relevant subset of features to improve computational efficiency or reduce the model’s generalisation error. SFFS starts with zero feature and finds the one that maximises a score when an estimator is trained on this single feature. This procedure is repeated until the total number of features is reached or there is no improvement. The score selected was given from a support vector machine (SVM) model [25, 26]. The objective of SVM is to maximise the margin between cases and controls, which is defined as the distance between the separating hyperplane (decision boundary) and the training samples that are closest to this hyperplane, as shown in Fig. 3.

### Statistical analysis

Data analysis and graph visualisation were performed using Matlab (version 2001b), R (version 4.1.2, R package: gplots package heatmap.2 function), and RStudio (version 2022.02.3) programs. We assessed the intercorrelation between conventional CMR metrics and radiomics features using Pearson’s correlation. Due to the large number of radiomics features, we grouped the inter-correlated variables into six clusters using hierarchical clustering, as per our previous publication [27].

We created hierarchical models to understand the influence of vascular risk factors (VRFs), conventional CMR indices and radiomics features, and their integrated use in the prediction of incident CVDs (AF, HF, MI, and stroke). The first three models assess the performance of VRF, conventional CMR indices, and CMR radiomics separately. Next, we combined categories as follows: VRF-CMR indices, VRF-radiomics, and CMR indices-radiomics. Finally, we merged all three components into an integrative model: VRF-CMR indices-radiomics. The summary of the process is shown in Fig. 2.

Training datasets are used to train and tune the parameters of the model, then a separate testing set is used to assess the performance of the model to see that the model built is able to generalise to unseen data. SVM is used for classification. We chose SVM due to its properties: good performance in real-world applications, computationally efficient, robust in high dimension, and sound in theoretical foundations. In order to tune the SVM parameters, brute force exhaustive search also known as greedy optimisation is used. The model is then trained with the parameters optimised. This procedure of tuning and training is performed five times each with different partitions of training (80%) and test (20%) samples to reduce overfitting. The average error of the testing folds determines the performance of the model.

We determined model performance using receiver operating characteristic (ROC) curve and area under the curve (AUC) scores. To assess the model accuracy, the mean accuracy, sensitivity, specificity, and AUC are reported. Welch’s *t*-test and chi-squared test were used for group-wise comparisons for continuous and categorical values, respectively.

## Results

### Baseline characteristics

The subjects’ characteristics are summarised in Table 1. CMR data was available for 32,121 UKB participants. For the whole imaging set, the average age was 63.3 (± 7.5) years, and the sample included 51.9% women. Over 3.7 (± 1.3) years of prospective follow-up, 193 participants had incident AF, 209 incident HF, 218 incident MI, and 199 incident stroke. Men were more likely to experience all incident CVDs considered. As expected, individuals who experienced incident CVD events had a greater overall risk factor burden.

**Table 1:** Baseline characteristics

Characteristics	Whole imaging set (*n* = 32,121)	Incident atrial fibrillation (*n* = 193)	Incident heart failure (*n* = 209)	Incident myocardial infarction (*n* = 218)	Incident stroke (*n* = 199)
Age mean (std)	63.3 (± 7*.*5)	66.9 (± 6.4)	68.7 (± 6.2)	66.2 (± 7.3)	67 (± 8)
Female sex, *n* (%)	16,658 (51.7%)	59 (30.6%)	72 (34.4%)	66 (30.3%)	75 (37.7%)
Townsend Deprivation Index, median (IQR)	−2.0 (3.3)	−2.6 (2.9)	−2.6 (2.9)	−2.5 (3.8)	−3.0 (2.5)
Body mass index, mean (kg/m^2^)	26.6 (± 4*.*4)	27.0 (± 4*.*4)	28.3 (± 4*.*9)	27.7 (± 4*.*0)	27.0 (± 3*.*5)
Current smoker, *n* (%)	2032 (6.3%)	13 (6.7%)*	15 (7.2%)*	20 (9.2%)*	11 (5.5%)*
Diabetes status, *n* (%)	993 (3.1%)	10 (5.2%)*	15 (7.2%)	11 (5.0%)*	8 (4.0%)*
Hypertension status, *n* (%)	4397 (13.7%)	54 (28.0%)	79 (37.8%)	60 (27.5%)	42 (21.1%)
High cholesterol status, *n* (%)	7272 (22.6%)	49 (25.4%)*	84 (40.2%)	64 (29.4%)	61 (30.7%)
IPAQ (MET minutes/week), median [IQR]	1528 [2360]	1519 [2892]*	1470 [2574]*	1281 [2262]*	1706 [2419]*
Education level, *n* (%)					
Left school age 14 or younger	421 (1.3%)	2 (1.0%)*	3 (1.4%)	2 (0.9%)	3 (1.5%) **
Left school age 15 or older	2260 (7.0%)	13 (6.7%)*	33 (15.8%)	26 (11.9%)	15 (7.5%)
High school diploma	4229 (13.2%)	32 (16.6%)*	37 (17.7%)	39 (17.9%)	24 (12.1%)
Sixth form qualification	1820 (5.7%)	11 (5.7%)*	11 (5.3%)	13 (6.0%)	12 (6.0%)
Professional qualification	8953 (27.9%)	64 (33.2%)*	57 (27.3%)	68 (31.2%)	52 (26.1%)
Higher education university degree	14,438 (44.9%)	71 (36.8%)*	68 (32.5%)	70 (32.1%)	93 (46.7%)
Alcohol intake, *n* (%)					
Never	1547 (4.8%)	14 (7.3%)	10 (4.8%) (**)	14 (6.4%) (**)	8 (4.0%) (**)
Special occasions only	2646 (8.2%)	9 (4.7%)	15 (7.2%)	20 (9.2%)	15 (7.5%)
1–3 times a month	3452 (10.7%)	17 (8.8%)	26 (12.4%)	26 (11.9%)	23 (11.6%)
1–2 times a week	8284 (25.8%)	37 (19.2%)	55 (26.3%)	51 (23.4%)	43 (21.6%)
3–4 times a week	9094 (28.3%)	65 (33.7%)	58 (27.8%)	61 (28.0%)	55 (27.6%)
Daily or almost daily	7098 (22.1%)	51 (26.4%)	45 (21.5%)	46 (21.1%)	55 (27.6%)
CMR indices mean (± std)					
LVEDVi, ml/m^2^	78.5 (± 14.2)	84.2 (± 21.7)	88.0 (± 24.9)	80.2 (± 13.9)*	81.3 (± 17.3)*
LVESVi, ml/m^2^	32.0 (± 8.8)	36.2 (± 16.1)	42.6 (± 21.0)	33.7 (± 10.3)	35.0 (± 12.0)
LVSVi, ml/m^2^	46.5 (± 8.5)	48.0 (± 11.7)	45.5 (± 11.0)	46.5 (± 8.4)*	46.4 (± 9.1)*
LVMi, g/m^2^	45.6 (± 8.9)	50.8 (± 11.8)	53.7 (± 14.7)	49.8 (± 9.4)	49.8 (± 10.6)
LVEF, %	59.5 (± 6.2)	57.8 (± 8.5)*	53.2 (± 10.3)	58.5 (± 7.6)*	57.6 (± 7.0)
RVEDVi, ml/m^2^	82.9 (± 15.5)	86.8 (± 17.9)	82.6 (± 17.8)*	82.6 (± 15.0)*	83.7 (± 15.1)*
RVESVi, ml/m^2^	35.7 (± 9.4)	38.9 (± 11.0)	37.7 (± 11.7)	36.1 (± 9.4)*	36.9 (± 9.4)*
RVSVi, ml/m^2^	47.2 (± 8.9)	47.9 (± 10.6)*	44.9 (± 10.2)	46.5 (± 8.8)*	46.9 (± 8.4)*
RVEF, %	57.2 (± 6.2)	55.5 (± 7.1)	54.7 (± 7.9)	56.6 (± 6.5)*	56.3 (± 5.8)

Abbreviations: *n*, number of cases; *IPAQ*, International Physical Activity Questionnaire; *METS*, metabolic equivalents; *EF*, ejection fraction; *EDV* end-diastolic volume; *ESV*, end-systolic volume; *LV*, left ventricle; *RV*, right ventricle; *SV*, stroke volume. “*” indicates no statistical differences between the whole population and the incident cardiovascular event using Welch’s *t*-test for continuous values and chi-squared test for categorical variables (*p* value > 0.05). “**” indicates no statistical difference for the categorical variables computed as a single groupwise for alcohol intake and education level variables

Conventional CMR metrics differed among at-risk groups and the whole imaging set: participants, who later developed AF, HF, MI, or stroke had on average higher LVMi (*p* < 0.05). The HF group had larger LVEDVi, and reduced LVEF (*p* < 0.05) compared to the whole imaging set.

### Correlation between CMR metrics and radiomics features

**Fig. 4:**
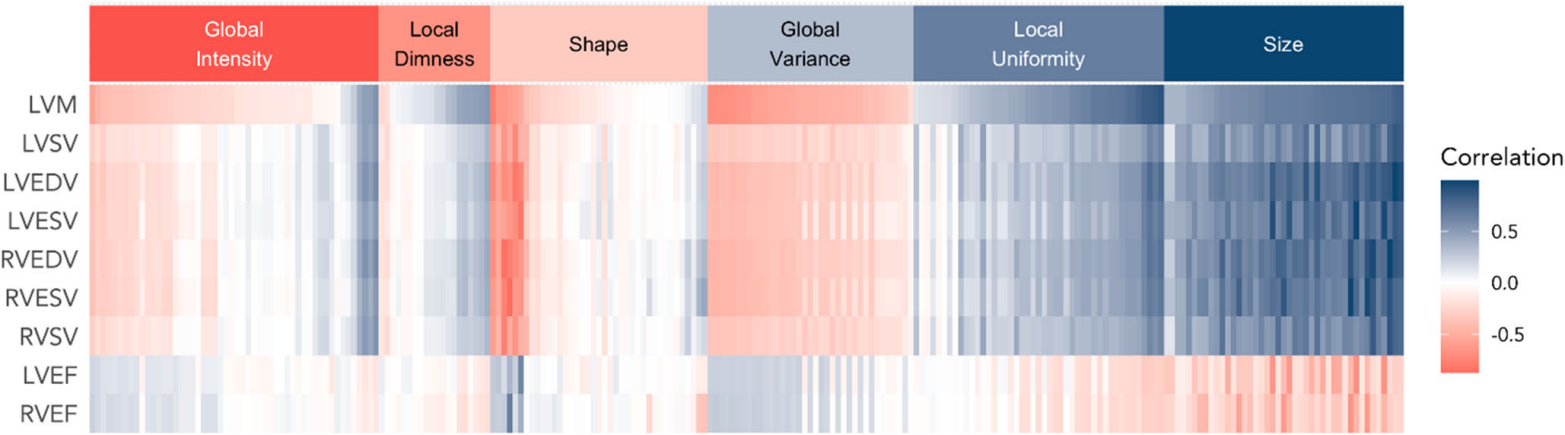
Correlation matrix of conventional CMR indices vs radiomics features in the whole sample. The correlation matrix illustrates correlation of each radiomics feature on the *x*-axis with the conventional CMR metrics indicated on the *y*-axis. Due to the large number of radiomics features, we grouped the inter-correlated variables into six clusters using hierarchical clustering using Ward’s algorithm. Abbreviations: LVEDV, left ventricular end-diastolic volume; LVEF, left ventricular ejection fraction; LVESV, left ventricular end-systolic volume; LVM, left ventricular mass; RVEDV, right ventricular end-diastolic volume; RVESV, right ventricular end-systolic volume; RVSV, right ventricular stroke volume

Figure 4 shows the correlation pattern between conventional CMR metrics and the imaging set’s radiomics features. Overall, size radiomics features showed the strongest correlation with conventional metrics. Moreover, some parameters from the local uniformity and shape groups also correlated with conventional metrics. Contrary to that, the majority of global intensity, local dimness, and global variance features showed inconsistent correlation patterns with CMR indices. Thus, although there is some overlap of conventional and radiomics CMR metrics, there are many areas where radiomics features provide new information.

### Identification of metrics for each CVD outcome

The features selected for each model are shown in Supplementary Tables 2, 3, 4, and 5. Feature importance is shown as the accuracy given by the SVM algorithm for each standalone feature.

The SFFS algorithm chose hypertension for all predictive models; its standalone accuracy was similar among incident outcomes, except for stroke which was lower (accuracy: AF vs HF vs MI vs Stroke — 0.59 vs 0.62 vs 0.58 vs 0.55). Sex was included in all but the HF models. LVM and LVSV were the two conventional features consistently selected by the SFFS. The accuracy of LVM alone was higher in all models compared to LVSV.

The identified radiomics signatures for each incident outcome are depicted in Table 2. Overall, ventricular shape and myocardial texture feature dominated all models and there was only a marginal role for first-order features. Indeed, HF and MI prediction models included only shape and texture features. Radiomics features derived from the LV blood pool and myocardium dominated all prediction models. Notably, when conventional CMR metrics and radiomics features were included alongside each other, the latter were selected more frequently than the former.

**Table 2:** Radiomics features selected for each incident CVD event

Incident cardiovascular outcome	Radiomics feature	Feature type	ROI	Phase	SVM model alone
Atrial fibrillation	Maximum 2D diameter slice	Shape	MYO	ES	0.67 (± 0.07)
	Energy	First-order	MYO	ES	0.57 (± 0.03)
	Maximum 2D diameter column	Shape	LV	ES	0.58 (± 0.01)
	Maximum 2D diameter row	Shape	MYO	ES	0.60 (± 0.07)
	Dependence non-uniformity	Texture	MYO	ES	0.65 (± 0.08)
	Inverse difference moment	Texture	MYO	ED	0.58 (± 0.06)
	Large area low grey level emphasis	Texture	MYO	ED	0.59 (± 0.06)
	Large area low grey level emphasis	Texture	MYO	ES	0.59 (± 0.03)
	Maximum 2D diameter row	Shape	LV	ES	0.56 (± 0.04)
	Surface area	Shape	LV	ES	0.63 (± 0.07)
	Maximum 2D diameter slice	Shape	LV	ED	0.62 (± 0.05)
	Maximum 3D diameter	Shape	MYO	ES	0.61 (± 0.05)
	Sum of squares	Texture	MYO	ES	0.55 (± 0.02)
	Zone variance	Texture	MYO	ED	0.64 (± 0.09)
	Maximum 2D diameter row	Shape	MYO	ED	0.58 (± 0.06)
	Energy	First-order	LV	ED	0.58 (± 0.03)
	Grey level non-uniformity	Texture	MYO	ES	0.65 (± 0.04)
	Run percentage	Texture	MYO	ED	0.60 (± 0.08)
	Major axis	Shape	MYO	ES	0.63 (± 0.06)
Heart failure	Maximum 2D diameter slice	Shape	MYO	ES	0.68 (± 0.06)
	Minor axis	Shape	LV	ES	0.66 (± 0.06)
	Volume	Shape	RV	ED	0.56 (± 0.05)
	Large area low grey level emphasis	Texture	MYO	ES	0.58 (± 0.02)
	Volume	Shape	LV	ES	0.64 (± 0.06)
	Informal measure of correlation	Texture	MYO	ED	0.57 (± 0.07)
	Small dependence emphasis	Texture	MYO	ED	0.52 (± 0.05)
	Grey level non-uniformity	Texture	MYO	ED	0.64 (± 0.07)
	Surface area	Shape	MYO	ED	0.63 (± 0.03)
Myocardial infarction	Coarseness	Texture	MYO	ES	0.64 (± 0.02)
	Maximum 2D diameter row	Shape	RV	ED	0.54 (± 0.05)
	Dependence variance	Texture	MYO	ES	0.52 (± 0.03)
	Inverse variance	Texture	MYO	ED	0.56 (± 0.02)
	Large area emphasis	Texture	MYO	ED	0.62 (± 0.02)
	Grey level variance	Texture	MYO	ED	0.52 (± 0.04)
	Sphericity	Shape	RV	ES	0.53 (± 0.04)
	Sphericity	Shape	MYO	ED	0.61 (± 0.02)
	Complexity	Texture	MYO	ES	0.56 (± 0.04)
Stroke	Surface area to volume ratio	Shape	MYO	ED	0.64 (± 0.02)
	Median	First-order	MYO	ES	0.57 (± 0.06)
	Busyness	Texture	MYO	ES	0.57 (± 0.04)
	Large area low grey level emphasis	Texture	MYO	ES	0.55 (± 0.04)
	Grey level non-uniformity	Texture	MYO	ES	0.63 (± 0.01)
	Root mean squared	First-order	MYO	ES	0.54 (± 0.05)
	Large area low grey level emphasis	Texture	MYO	ED	0.57 (± 0.04)
	Mean	First-order	MYO	ES	0.55 (± 0.06)
	Large dependence low grey level emphasis	Texture	MYO	ED	0.57 (± 0.04)
	Sphericity	Shape	LV	ED	0.52 (± 0.05)
	Contrast	Texture	MYO	ED	0.56 (± 0.03)
	Grey level non-uniformity	Texture	MYO	ES	0.61 (± 0.01)
	Difference entropy	Texture	MYO	ED	0.57 (± 0.04)
	Energy	First-order	MYO	ES	0.48 (± 0.03)
	Sphericity	Shape	MYO	ED	0.59 (± 0.04)
	Joint average	Texture	MYO	ES	0.56 (± 0.05)
	Range	First-order	MYO	ED	0.56 (± 0.07)
	Large area emphasis	Texture	MYO	ED	0.60 (± 0.01)
	Sum entropy	Texture	MYO	ES	0.54 (± 0.02)

Abbreviations: *ROI*, region of interest; *SVM model alone*, support vector machine model performance showing the mean and standard deviation using each radiomics feature individually; *LV*, left ventricle; *RV*, right ventricle; *MYO*, left ventricle myocardium; *ED*, end diastolic

Shape features depicting the “maximum diameter” presented the most discriminative power in AF, alongside texture features of non-uniformity. In the HF model, shape features (maximum diameter, minor axis, and volume) presented the greatest selective power, whilst in the MI model, the texture features, such as coarseness or large area emphasis, were more prominent.

### The degree of discrimination achieved for each incident CVD

**Fig. 5:**
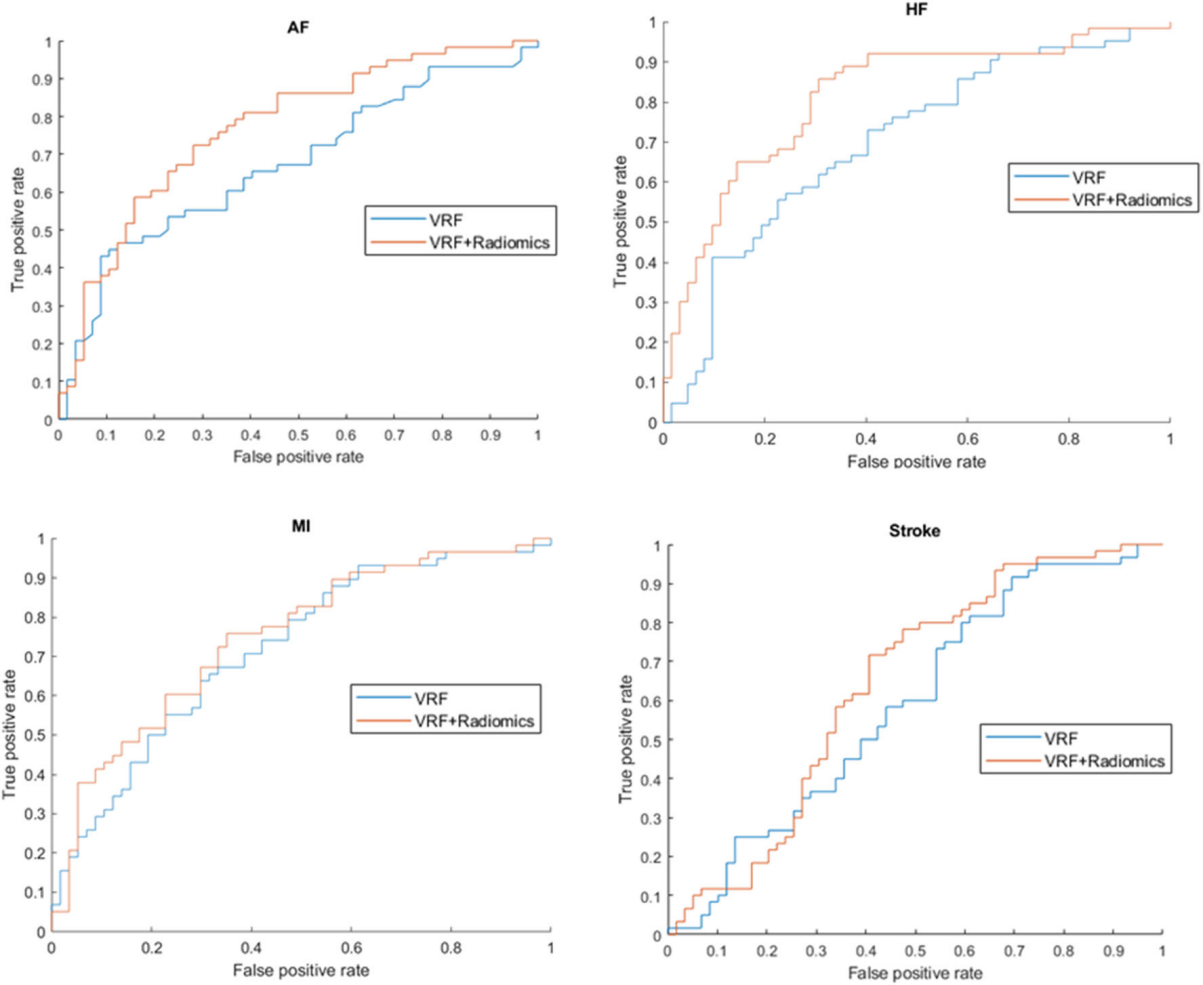
ROC curves showing the discriminative power of vascular risk factors alone and the combination of vascular risk factors and radiomics feature in all incident outcome prediction model. The combination of vascular risk factors (VRFs) and radiomics features (orange) reached better performance in the prediction of AF and HF compared to VRF alone (blue) (*p* < 0.05). Abbreviations: AF, atrial fibrillation; HF, heart failure; MI, myocardial infarction

Results from the hierarchical models are summarised in Table 3. The average error of the testing folds determines the performance of the model. Radiomics models alone yielded slightly better discrimination and higher sensitivity than VRFs or conventional CMR models in each outcome. AF and HF prediction models performed generally better than MI and stroke prediction models. The addition of radiomics features improved the performance of VRF models in AF (AUC: 0.67 vs 0.76) and HF (AUC: 0.73 vs 0.83) prediction (Fig. 5).

**Table 3:** The performance of all the models computing the average and standard deviation of accuracy, sensitivity, specificity, and AUC of 5 different test folds

		VRF	CMR	Radiomics	VRF + CMR	VRF + radiomics	CMR + radiomics	VRF + CMR + radiomics
AF	Accuracy	0.67 (± 0.03)	0.66 (± 0.03)	0.68 (± 0.05)	0.67 (± 0.04)	0.69 (± 0.06)	0.70 (± 0.07)	0.71 (± 0.08)
	Sensitivity	0.69 (± 0.04)	0.68 (± 0.02)	0.77 (± 0.06)	0.68 (± 0.1)	0.73 (± 0.07)	0.76 (± 0.1)	0.72 (± 0.1)
	Specificity	0.64 (± 0.05)	0.63 (± 0.09)	0.60 (± 0.06)	0.64 (± 0.05)	0.70 (± 0.08)	0.66 (± 0.03)	0.70 (± 0.08)
	AUC	0.67 (± 0.05)	0.68 (± 0.04)	0.73 (± 0.06)	0.67 (± 0.06)	0.76 (± 0.06)	0.73 (± 0.07)	0.76 (± 0.07)
HF	Accuracy	0.66 (± 0.03)	0.70 (± 0.02)	0.71 (± 0.03)	0.74 (± 0.02)	0.77 (± 0.02)	0.70 (± 0.06)	0.79 (± 0.02)
	Sensitivity	0.63 (± 0.04)	0.61 (± 0.01)	0.82 (± 0.06)	0.80 (± 0.06)	0.74 (± 0.06)	0.63 (± 0.08)	0.73 (± 0.04)
	Specificity	0.69 (± 0.06)	0.82 (± 0.05)	0.65 (± 0.05)	0.66 (± 0.06)	0.79 (± 0.04)	0.75 (± 0.1)	0.85(± 0.03)
	AUC	0.73 (± 0.03)	0.74 (± 0.02)	0.75 (± 0.02)	0.82 (± 0.03)	0.83 (± 0.03)	0.76 (± 0.8)	0.84 (± 0.02)
MI	Accuracy	0.67 (± 0.02)	0.67 (± 0.02)	0.70 (± 0.06)	0.69 (± 0.01)	0.67 (± 0.05)	0.67 (± 0.05)	0.71(± 0.04)
	Sensitivity	0.69 (± 0.06)	0.58 (± 0.08)	0.75 (± 0.05)	0.70 (± 0.05)	0.69 (± 0.04)	0.64 (± 0.07)	0.76 (± 0.05)
	Specificity	0.58 (± 0.03)	0.75 (± 0.04)	0.64 (± 0.1)	0.66 (± 0.04)	0.66 (± 0.06)	0.73 (± 0.08)	0.65 (± 0.05)
	AUC	0.70 (± 0.03)	0.73 (± 0.04)	0.75 (± 0.04)	0.73 (± 0.03)	0.72 (± 0.04)	0.71 (± 0.04)	0.76 (± 0.04)
Stroke	Accuracy	0.58 (± 0.03)	0.61 (± 0.01)	0.64 (± 0.03)	0.65 (± 0.04)	0.63 (± 0.03)	0.64 (± 0.03)	0.64 (± 0.03)
	Sensitivity	0.63 (± 0.03)	0.60 (± 0.04)	0.81 (± 0.05)	0.61 (± 0.02)	0.51 (± 0.07)	0.81 (± 0.05)	0.74 (± 0.06)
	Specificity	0.52 (± 0.03)	0.62 (± 0.06)	0.45 (± 0.03)	0.69 (± 0.03)	0.74 (± 0.03)	0.45 (± 0.03)	0.64 (± 0.03)
	AUC	0.58 (± 0.02)	0.65 (± 0.03)	0.68 (± 0.04)	0.61 (± 0.04)	0.63 (± 0.04)	0.68 (± 0.04)	0.63 (± 0.05)

Abbreviations: *CMR*, cardiac magnetic resonance; *VRF*, vascular risk factor, *AF*, atrial fibrillation, *HF*, heart failure; *MI*, myocardial infarction

Moreover, VRFs and radiomics features’ combination reached better performance than VRFs and conventional CMR metrics in AF, HF, and stroke prediction models. We reached the best performance in the incident AF prediction model combining VRFs, CMR indices, and radiomics features (Table 3).

In Supplementary Table 6, we have added an additional experiment defining the healthy controls as subjects not having any cardiovascular disease or stroke at the baseline visit and during follow-up to see if the models behave in the same way. The results followed the same pattern for all the models except in the sensitivity which was lower. Additionally, the models stabilised with 40 features in the univariate feature selection. We could conclude that the performance of our model is rather similar regardless of the comparator groups, suggesting that the patterns we pick up are stable.

## Discussion

In this study, we demonstrate the feasibility of CMR-derived radiomics features to predict incident AF, HF, MI, and stroke. Additionally, using hierarchically built SVM models, we demonstrate the incremental value of CMR radiomics features for risk prediction over VRFs and conventional CMR metrics.

### Comparison with existing literature

To the best of our knowledge, this is the first study to demonstrate the value of CMR radiomics models for incident CVD prediction. Previous research supports the utility of CMR radiomics in the differential diagnosis of left ventricular hypertrophy [28], especially the diagnosis of hypertrophic cardiomyopathy (HCM) [12, 29, 30]. Cetin et al have shown the technique’s potential to identify imaging signatures associated with cardiovascular risk factors such as diabetes or hypertension [13]. Furthermore, Raisi-Estabragh et al demonstrated the independent associations of CMR phenotypes with sex, age, and important VRFs [27]. Recently, Ma et al concluded that a non-contrast T1 map–based radiomics nomogram is suitable for predicting major adverse cardiac events in patients with acute MI [31].

We built hierarchical models to test the utility and added benefit of including radiomics features in predicting AF, HF, MI, and stroke using the SFFS algorithm. Not surprisingly, hypertension proved a crucial predisposing factor linked to all considered outcomes. This finding is consistent with the overwhelming evidence showing that among all risk factors for CVD, hypertension is associated with the strongest causal link to adverse outcomes [32–36]. Sex was selected for inclusion in all predictive models, except for HF, a finding that is in line with the results from major epidemiological studies [37, 38] showing that the lifetime risk of HF is comparable among males and females. Of note, we did not differentiate subgroups of HF, which clearly show sex-specific differences as emphasised by Lam et al [39]. Left ventricular hypertrophy (most commonly assessed by LVM increase) is a remarkable prognostic marker that incorporates a broad range of pathologies, such as hypertrophic and infiltrative cardiomyopathies, although it is most commonly caused by chronic pressure and volume overload [40]. Early studies have recognised increased LVM as a risk factor for stroke in the Framingham Heart Study [41]. LVM has been widely utilised ever since due to its ability to predict a variety of clinical outcomes [40]. Whilst conventional metrics quantify LVM according to mass or wall thickness, radiomics analysis can additionally quantify the distribution and pattern of myocardial signal intensities within the LV myocardium. As such, radiomics features extracted from the myocardium may provide more granular distinction of health and disease in comparison to conventional CMR indices where, rather crudely, the single most discriminatory feature for all risk factors was higher LVM [13]. Indeed, Schofield et al showed that texture radiomics features derived from bSSFP sequences can differentiate between the aetiologies of LV hypertrophy [42]. These findings suggest that radiomics has the capability to enrich risk information beyond the limits of LVM. In our study, texture features were identified as the most defining model predictors, highlighting the clinical relevance of these metrics.

Finally, we illustrated that radiomics features derived from CMR could provide incremental discriminative value over VRFs and CMR indices in the prediction of incident AF and HF. The HF model showed the most robust improvement with the addition of radiomics features, whilst stroke prediction showed only a slight improvement in the hierarchical models. This might be partially due to the aetiology: diseases such as dilated cardiomyopathy (the most common non-ischaemic cause of HF [30]) that primarily affect the global muscular structure of the heart may be better captured by CMR radiomics. In contrast, MI typically comprises more focal areas of myocardial injury and stroke is a primary cerebral illness.

### Clinical interpretation of radiomics findings

Shape features and texture radiomics features presented the most discriminative value in AF prediction models. The most prominent shape feature was the maximum diameters of the LV and the ventricular wall in different phases of the cardiac cycle. This refers to the notion that the adverse remodelling of the heart described by larger chamber sizes and hypertrophy predispose AF. Alterations of the non-uniformity levels (“dependence non-uniformity” and “grey level non-uniformity”) are referring to changes in the heterogeneity of intensity values, which might reflect on the adverse changes in tissue composition of the myocardial structure. Similarly, “large area low grey level emphasis” suggests larger myocardial regions with low signal intensity (dimmer) pixels. Indeed, LV diastolic dysfunction has been linked to an increased risk of AF in the general population [43], and more recently Tian et al demonstrated the association between adverse LV remodelling and AF among HCM patients [44].

In the HF models, shape features, derived from the myocardium, LV, and RV demonstrated strong discriminatory value. This can be explained by adverse and often biventricular remodelling that characterises HF patients. Our results suggested that apart from the diameter of a given slice, the elongation of the heart (depicted by “minor axis”) also provides additional information.

### Limitations

Although our analysis is performed with different partitions of data to have a model independent to the samples by minimising the case of over-fitting, the model might still be biased to the participants obtained in the UKB. In this proof-of-concept study, we limited our investigations to LV and RV metrics derived from bSSFP images. The clinical utility of this proof-of-concept study is limited in its current state: (1) CMR is not a routine examination; (2) CMR should not be performed for the sole purpose of risk stratification. However, we believe it is reasonable to postulate that the radiomics models may be a useful enhancement to existing CMR scans performed with a clinical indication and may improve risk stratification in the future.

Moreover, no external validation has been performed, and the case-control design leaves significant risk of residual confounding. Of note, only 5% of the UK Biobank population was studied and a 2.5% event rate in this hypothesis generating study. Thus, the predictiveness of the model if these radiomic metric were deployed in the general cohort remains unanswered.

## Conclusions

We demonstrated the feasibility of using CMR-derived radiomics features to predict key cardiovascular outcomes. Radiomics features provided additional information over VRFs, although the improvement was only marginal compared to conventional CMR metrics. The improvement was most prominent in AF and HF prediction, which highlight that the performance of radiomics models is dependent on the disease aetiology and mechanism.

## Supplementary Information

ESM 1 (DOCX 95.4 kb)

## Abbreviations

AF: Atrial fibrillation
AUC: Area under the curve
CMR: Cardiovascular magnetic resonance imaging
CVD: Cardiovascular disease
ED: End diastole
EF: Ejection fraction
ES: End systole
HES: Hospital Episode Statistics
HF: Heart failure
ICD: International Classification of Diseases
LV: Left ventricle
M: Mass
MI: Myocardial infarction
ML: Machine learning
MYO: Myocardium
NHS: National Health Service
Rad: Radiomics
ROC: Receiver operating characteristic
ROI: Region of interest
RV: Right ventricle
SFFS: Sequential feature forward selection
SV: Stroke volume
SVM: Support vector machine
UKB: UK Biobank
V: Volume
VRF: Vascular risk factor

## Declarations

### Guarantor

The scientific guarantor of this publication is Dr. Karim Lekadir.

### Statistics and biometry

Zahra Raisi-Estabragh and Karim Lekadir kindly provided statistical advice for this manuscript.

### Study subjects or cohorts overlap

Some study subjects or cohorts have been previously reported in literature as the UKB is widely used for research. But the study is completely new.

### Methodology

• prospective

• diagnostic or prognostic study

• performed at one institution
