# Supplementary material for: Prediction of incident cardiovascular events using machine learning and CMR radiomics
Source: Eur Radiol. 2022 Dec 13;33(5):3488–500. doi: 10.1007/s00330-022-09323-z (PMC10121487; doi:10.1007/s00330-022-09323-z)
Supplement: Supplementary file 1 — (DOCX 95.4 kb) [file 330_2022_9323_MOESM1_ESM.docx]

***Supplementary Table 1: Disease definitions.***

| **Source** | **UKB Field  ID: code** | **Description** |
| --- | --- | --- |
| **Myocardial infarction** | | |
| Self-report | 20002 | Heart attack/myocardial infarction |
| Algorithm | 42000 | Date of myocardial infarction |
| ICD10 | I21 | Acute myocardial infarction |
|  | I22 | Subsequent myocardial infarction |
|  | I23 | Certain current complications following acute myocardial infarction |
| First occurrences | 131298 | Acute myocardial infarction |
|  | 131300 | Subsequent myocardial infarction |
|  | 131302 | Certain current complications following acute myocardial infarction |
| Diagnosed by doctor | 6150: 1 | Heart attack |
|  | 3894 | Age heart attack diagnosed |
| ICD9 | 410 | Acute myocardial infarction |
|  | 411 | Other acute and subacute forms of ischaemic heart disease |
|  | 412 | Old myocardial infarction |
| **Heart failure** | | |
| Self-report | 20002 | Heart failure/pulmonary odema |
| ICD10 | I500 | Congestive heart failure |
|  | I501 | Left ventricular failure |
|  | I509 | Heart failure, unspecified |
| First occurrences | 131354 | heart failure |
| **Atrial fibrillation** | | |
| Self-report | 20002 | Atrial fibrillation |
| ICD10 | I480 | Paroxysmal atrial fibrillation |
|  | I481 | Persistent atrial fibrillation |
|  | I482 | Chronic atrial fibrillation |
| **Stroke** |  |  |
| Self-report | 20002 | Stroke |
|  | 20002 | Ischaemic stroke |
|  | 20002 | Brain haemorrhage |
| Algorithm | 42006 | Date of stroke |
|  | 42008 | Date of ischaemic stroke |
|  | 42010 | Date of intracerebral haemorrhage |
| Diagnosed by doctor | 6150: 3 | Stroke |
|  | 4056 | Age stroke diagnosed |
| ICD10 | I61 | Intracerebral haemorrhage |
|  | I62 | Other nontraumatic intracranial haemorrhage |
|  | I63 | Cerebral infarction |
|  | I64 | Stroke, not specified as haemorrhage or infarction |
| ICD9 | 431 | Intracerebral haemorrhage |
|  | 432 | Other and unspecified intracranial haemorrhage |
|  | 434 | Occlusion of cerebral arteries |
|  | 436 | Acute but ill-defined cerebrovascular disease |
| First occurrences | 131362 | Intracerebral haemorrhage |
|  | 131364 | Other nontraumatic intracranial haemorrhage |
|  | 131366 | Cerebral infarction |
|  | 131368 | Stroke, not specified as haemorrhage or infarction |
| **Diabetes** |  |  |
| Diagnosed by doctor | 2443 | Diabetes diagnosed by doctor |
|  | 2976 | Age diabetes diagnosed by doctor |
| Medications | 6177, 6153: 3 | Insulin |
| Biochemistry | 30750 | Glycated haemoglobin (HbA1c) > 48 mmol/mol |
| **High cholesterol** |  |  |
| Medications | 6177, 6153: 1 | Cholesterol lowering medication |
| Biochemistry | 30690 | Cholesterol > 7 mmol/L |
| **Hypertension** |  |  |
| Medications | 6177, 6153: 2 | Blood pressure medication |

ICD10 codes are drawn from fields 41270, 41280, 41234 and 41259

ICD9 codes are drawn from fields 41271, 41281, 41234 and 41259

Where a 3-digit code is given, this includes all 4-digit sub-codes, for example, I21 includes I210, I211 and I212 etc.

**Supplementary Table 2: Atrial fibrillation**

| AF Model | Features Selected (ordered as selected) | Type | ROI | Phase | SVM alone |
| --- | --- | --- | --- | --- | --- |
| VRF | Sex  Age  Hypertension |  |  |  | 0.66(±0.05)  0.59(±0.04)  0.59(±0.06) |
| CMR | LVM  RVEF  LVEDV  LVSV |  |  |  | 0.66(±0.07)  0.59(±0.03)  0.59 (±0.02)  0.56 (±0.05) |
| CMR+VRF | LVM  RVEF  Hypertension  LVSV  Age  RVEDV  LVEDV |  |  |  | 0.66 (±0.07)  0.59 (±0.03)  0.59 (±0.06)  0.56 (±0.05)  0.59 (±0.04)  0.62 (±0.03)  0.59 (±0.02) |
| Radiomics | Maximum 2D diameter slice  Energy  Maximum 2D diameter column  Maximum 2D diameter row  Dependence non-Uniformity  Inverse difference moment  Large area low gray level emphasis  Large area low gray level emphasis  Maximum 2D Diameter row  Surface area  Maximum 2D diameter slice  Maximum 3D diameter  Sum of squares  Zone variance  Maximum 2D diameter row  Energy  Gray level non-uniformity glrm  Run percentage  Major axis | Shape  First-Order  Shape  Shape  Texture  Texture  Texture  Texture  Shape  Shape  Shape  Shape  Texture  Texture  Shape  First-Order  Texture  Texture  Shape | MYO  MYO  LV  MYO  MYO  MYO  MYO  MYO  LV  LV  LV  MYO  MYO  MYO  MYO  LV  MYO  MYO  MYO | ES  ES  ES  ES  ES  ED  ED  ES  ES  ES  ED  ES  ES  ED  ED  ED  ES  ED  ES | 0.67 (±0.07)  0.57 (±0.03)  0.58 (±0.01)  0.60 (±0.07)  0.65 (±0.08)  0.58 (±0.06)  0.59 (±0.06)  0.59 (±0.03)  0.56 (±0.04)  0.63 (±0.07)  0.62 (±0.05)  0.61 (±0.05)  0.55 (±0.02)  0.64 (±0.09)  0.58 (±0.06)  0.58 (±0.03)  0.65 (±0.07)  0.60 (±0.08)  0.63 (±0.06) |
| Radiomics+VRF | Maximum 2D diameter slice  Age  Small area low gray level emphasis  Long run low gray level emphasis  Percentile 90th  Gray level non-uniformity glszm  Long run high gray level emphasis  Hypertension  Gray level non-uniformity normalized glrlm  Large area low gray level emphasis | Shape  Texture  Texture  First-Order  Texture  Texture  Texture  Texture | MYO  MYO  MYO  MYO  MYO  MYO  MYO  MYO | ES  ES  ES  ES  ES  ED  ES  ES | 0.67 (± 0.07)  0.59 (±0.04)  0.48 (±0.04)  0.55 (±0.02)  0.54 (±0.06)  0.58 (±0.04)  0.52 (±0.04)  0.59 (±0.06)  0.51 (±0.02)  0.59 (±0.03) |
| CMR+Radiomics | Maximum 2D diameter slice  Energy  Maximum 2D diameter column  RVEF  Gray level non-uniformity gldm  Maximum 3D diameter  Maximum 2D diameter column  Percentile 90th  Volume  LVM  Contrast  Sphericity  Major axis  Maximum 2D diameter slice | Shape  First-Order Shape  Texture  Shape  Shape  First-Order Shape  Texture  Shape  Shape  Shape | MYO  MYO  LV  MYO  MYO  LV  MYO  LV  MYO  RV  MYO  LV | ES  ES  ES  ES  ES  ED  ES  ES  ED  ES  ES  ED | 0.67 (±0.07)  0.57 (±0.03)  0.58 (±0.01)  0.59 (±0.03)  0.66 (±0.04)  0.61 (±0.05)  0.59 (±0.04)  0.54 (±0.06)  0.62 (±0.06)  0.66 (±0.07)  0.59 (±0.05)  0.53 (±0.03)  0.63 (±0.06)  0.62 (±0.05) |
| CMR+Radiomics+VRF | Maximum 2D diameter slice  Age  Small area low gray level emphasis  Long run low gray level emphasis  Percentile 90th  Gray level non-uniformity  Long run high gray level emphasis  Hypertension  Gray level non-uniformity normalized  Large area low gray level emphasis | Shape  Texture  Texture  Energy  Texture  Texture  Texture  Texture | MYO  MYO  MYO  MYO  MYO  MYO  MYO  MYO | ES  ES  ES  ES  ES  ED  ES  ES | 0.67 (±0.07)  0.59 (±0.04)  0.48(±0.04)  0.55 (±0.02)  0.54 (±0.06)  0.58 (±0.04)  0.52 (±0.04)  0.59(±0.06)  0.51 (±0.02)  0.59 (±0.03) |

*Abbreviations: CMR, cardiac magnetic resonance imaging; VRF, vascular risk factor, ROI, region of interest, SVM model alone: support vector machine model performance showing the mean and standard deviation using each radiomic feature individually; LV, left-ventricle; RV, right-ventricle; MYO, left ventricle myocardium; ED, end-diastolic, EF, ejection fraction, EDV end-diastolic volume, ESV, end-systolic volume, LV, left ventricle, RV right ventricle, SV stroke volume.*

**Supplementary Table 3: Heart Failure**

| HF Model | Features Selected (ordered as selected) | Type | ROI | Phase | SVM alone |
| --- | --- | --- | --- | --- | --- |
| VRF | Age  Body surface area  Hypertension  Diabetes  High cholesterol  Body mass index |  |  |  | 0.65(±0.08)  0.61 (±0.03)  0.62(±0.04)  0.53 (±0.02)  0.52(±0.06)  0.61(±0.06) |
| CMR | LVESV  LVM  RVEDV  LVEF  LVSV  RVEF |  |  |  | 0.66 (±0.05)  0.64 (±0.05)  0.57 (±0.05)  0.66 (±0.06)  0.52(±0.03)  0.61(±0.04) |
| CMR+ VRF | LVESV  Age  Body mass index  Body surface area  Hypertension  Diabetes |  |  |  | 0.66 (±0.05)  0.65 (±0.08)  0.61 (±0.06)  0.61 (±0.03)  0.62(±0.04)  0.53(±0.02) |
| Radiomics | Maximum 2D diameter slice  Minor axis  Volume  Large area low gray level emphasis  Volume  Informal measure of correlation1  Small dependence emphasis  Gray level non-uniformity  Surface area | Shape  Shape  Shape  Texture  Shape  Texture  Texture  Texture  Shape | MYO  LV  RV  MYO  LV  MYO  MYO  MYO  MYO | ES  ES  ED  ES  ES  ED  ES  ED  ED | 0.68 (±0.06)  0.66 (±0.06)  0.56 (±0.05)  0.58 (±0.02)  0.64 (±0.06)  0.57 (±0.07)  0.52 (±0.05)  0.64(±0.07)  0.63(±0.03) |
| Radiomics+VRF | Maximum 2D diameter slice  Minor axis  Age  Hypertension  Major axis  Size zone non-uniformity normalized  Least axis  Least axis | Shape  Shape  Shape  Texture  Shape  Shape | MYO  LV  RV  MYO  RV  LV | ES  ES  ED  ED  ED  ED | 0.68 (±0.06)  0.66 (±0.06)  0.65(±0.08)  0.62(±0.04)  0.51(±0.05)  0.53(±0.06)  0.52(±0.03)  0.60(±0.05) |
| CMR+Radiomics | Maximum 2D diameter slice  Minor axis  LVSV  Dependence non-uniformity | Shape  Shape  Texture | MYO  LV  MYO | ES  ES  ED | 0.68 (±0.06)  0.66 (±0.06)  0.52(±0.03)  0.62(±0.05) |
| CMR+Radiomics+VRF | Maximum 2D diameter slice  Minor axis  LVSV  Dependence non-uniformity  Hypertension  Mean absolute deviation  Run length non-uniformity  Age  Complexity  Low gray level zone emphasis | Shape  Shape  Texture  First-Order  Texture  Texture  Texture | MYO  LV  MYO  MYO  MYO  MYO  MYO | ES  ES  ED  ES  ES  ES  ED | 0.68 (±0.06)  0.66 (±0.06)  0.52(±0.03)  0.62 (±0.05)  0.62(±0.04)  0.54 (±0.03)  0.63 (±0.04)  0.65 (±0.08)  0.54(±0.04)  0.53(±0.04) |

*Abbreviations: CMR, cardiac magnetic resonance imaging; VRF, vascular risk factor, ROI, region of interest, SVM model alone: support vector machine model performance showing the mean and standard deviation using each radiomic feature individually; LV, left-ventricle; RV, right-ventricle; MYO, left ventricle myocardium; ED, end-diastolic, EF, ejection fraction, EDV end-diastolic volume, ESV, end-systolic volume, LV, left ventricle, RV right ventricle, SV stroke volume.*

**Supplementary Table 4: Myocardial infarction.**

| MI Model | Features Selected (ordered as selected) | Type | ROI | Phase | SVM alone |
| --- | --- | --- | --- | --- | --- |
| VRF | Sex  Hypertension  Body Surface Area |  |  |  | 0.66 (±0.04)  0.58 (±0.03)  0.54 (±0.02) |
| CMR | LVM  LVSV  RVEDV  RVESV |  |  |  | 0.65 (±0.02)  0.54 (±0.06)  0.55 (±0.06)  0.57 (±0.04) |
| CMR+ VRF | Sex  LVEDV  Body mass index  LVESV  Hypertension  Body Surface Area  LVSV |  |  |  | 0.66 (±0.04)  0.56 (±0.05)  0.61 (±0.03)  0.59 (±0.02)  0.58 (±0.03)  0.54 (±0.02)  0.54 (±0.06) |
| Radiomics | Coarseness  Maximum 2D diameter row  Dependence variance  Inverse variance  Large area emphasis  Gray level variance  Sphericity  Sphericity  Complexity | Texture  Shape  Texture  Texture  Texture  Texture  Shape  Shape  Texture | MYO  RV  MYO  MYO  MYO  MYO  RV  MYO  MYO | ES  ED  ES  ED  ED  ED  ES  ED  ES | 0.64 (±0.02)  0.54 (±0.05)  0.52 (±0.03)  0.56 (±0.02)  0.62 (±0.02)  0.52 (±0.04)  0.53 (±0.04)  0.61 (±0.02)  0.56 (±0.04) |
| Radiomics+VRF | Sex  Small dependence low gray level emphasis  Hypertension  Body Surface Area  Maximum 2D diameter slice  Dependence non uniformity  Max 2D diameter slice  Zone entropy  Maximum | Texture  Shape  Texture  Shape  Texture  First-Order | MYO  MYO  MYO  LV  MYO  MYO | ED  ED  ES  ES  ED  ES | 0.66 (±0.04)  0.53 (±0.06)  0.58 (±0.03)  0.54 (±0.02)  0.60 (±0.05)  0.64 (±0.02)  0.58 (±0.04)  0.54 (±0.06)  0.53 (±0.02) |
| CMR+Radiomics | LVM  Least axis  Major axis  Maximum 3D diameter  Surface area  Maximum 2D diameter row  Gray level non-uniformity normalized  Large area high gray level emphasis  Maximum 2D diameter row  Volume  Maximum 2D diameter slice  Surface area | Shape  Shape  Shape  Shape  Shape  Texture  Texture  Shape  Shape  Shape  Shape | RV  MYO  RV  MYO  MYO  MYO  MYO  MYO  MYO  MYO  RV | ED  ES  ED  ES  ES  ED  ES  ED  ES  ES  ED | 0.65 (±0.02)  0.54 (±0.04)  0.59 (±0.03)  0.60 (±0.05)  0.61 (±0.02)  0.57 (±0.01)  0.57 (±0.03)  0.58 (±0.02)  0.57 (±0.02)  0.63 (±0.03)  0.64(±0.05)  0.54 (±0.03) |
| CMR+Radiomics+  VRF | Sex  Small dependence low gray level emphasis  Hypertension  Maximum 2D diameter slice Dependence non-uniformity  Maximum 2D diameter slice  Zone Entropy  Maximum | Texture  Shape  Texture  Shape  Texture  First-Order | MYO  MYO MYO LV MYO MYO | ED  ED ES ES ED ES | 0.66 (±0.04)  0.53 (±0.06)  0.58 (±0.03)  0.60 (±0.05)  0.64 (±0.02)  0.58 (±0.04)  0.54 (±0.06)  0.53 (±0.02) |

*Abbreviations: CMR, cardiac magnetic resonance imaging; VRF, vascular risk factor, ROI, region of interest, SVM model alone: support vector machine model performance showing the mean and standard deviation using each radiomic feature individually; LV, left-ventricle; RV, right-ventricle; MYO, left ventricle myocardium; ED, end-diastolic, EF, ejection fraction, EDV end-diastolic volume, ESV, end-systolic volume, LV, left ventricle, RV right ventricle, SV stroke volume.*

**Supplementary Table 5: Stroke**

| Stroke Model | Features Selected (ordered as selected) | Type | ROI | Phase | SVM alone |
| --- | --- | --- | --- | --- | --- |
| VRF | Sex  Body surface area  Hypertension  Age |  |  |  | 0.62 (±0.03)  0.58 (±0.05)  0.55 (±0.03)  0.61 (±0.05) |
| CMR | LVM  RVSV  LVSV  LVEDV  LVESV |  |  |  | 0.61 (±0.02)  0.52 (±0.03)  0.51 (±0.04)  0.52 (±0.05)  0.55 (±0.03) |
| CMR+ VRF | Sex  LVM  LVSV  Hypertension  Age  RVEDV |  |  |  | 0.62 (±0.03)  0.61 (±0.01)  0.51(±0.04)  0.55 (±0.03)  0.61 (±0.05)  0.55 (±0.05) |
| Radiomics | Surface area to volume ratio  Median  Busyness  Large area low gray level emphasis  Gray level non-uniformity  Root mean squared  Large area low gray level emphasis  Mean  Large dependence low gray level emphasis  Sphericity  Contrast  Gray level non-uniformity  Difference entropy  Energy  Sphericity  Joint average  Range  Large area emphasis  Sum entropy | Shape  First-Order  Texture  Texture  Texture  First-Order Texture  First-Order  Texture  Shape  Texture  Texture  Texture  First-Order  Shape  Texture  First-Order  Texture  Texture | MYO  MYO  MYO  MYO  MYO  MYO  MYO  MYO  MYO  LV  MYO  MYO  MYO  MYO  MYO  MYO  MYO  MYO  MYO | ED  ES  ES  ES  ES  ES  ED  ES  ED  ED  ED  ES  ED  ES  ED  ES  ED  ED  ES | 0.64 (±0.02)  0.57 (±0.06)  0.57 (±0.04)  0.55 (±0.04)  0.63 (±0.01)  0.54 (±0.05)  0.57 (±0.04)  0.55 (±0.06)  0.57 (±0.04)  0.52 (±0.05)  0.56 (±0.03)  0.61 (±0.01)  0.57 (±0.04)  0.48(±0.03)  0.59 (±0.04)  0.56 (±0.05)  0.56(±0.07) 0.60(±0.01)  0.54(±0.02) |
| Radiomics+VRF | Surface area to volume ratio  Median  Busyness  Age  Large Area high gray level emphasis  Mean  Zone variance  Large area emphasis Zone variance  Sex  Busyness  Gray level non-uniformity  Root mean squared  Hypertension  Maximum 2D diameter slice  Short run low gray level emphasis  Long run high gray level emphasis  Low gray level emphasis | Shape  First-Order  Texture  Texture  First-Order  Texture  Texture  Texture  Texture  Texture  First-Order  Shape  Texture  Texture  Texture | MYO  MYO  MYO  MYO  MYO  MYO  MYO  MYO  MYO  MYO  MYO  LV  MYO  MYO  MYO | ED  ES  ES  ES  ED  ED  ED  ES  ED  ED  ED  ED  ED  ES  ED | 0.64 (±0.02)  0.57 (±0.06)  0.57 (±0.04)  0.61(±0.05)  0.55 (±0.04)  0.54 (±0.05)  0.60 (±0.01)  0.60 (±0.01)  0.59 (±0.05)  0.62 (±0.03)  0.57 (±0.04)  0.59 (±0.03)  0.56 (±0-05)  0.55 (±0.03)  0.55 (±0.04)  0.45(±0.03)  0.46(±0.05)  0.53(±0.03) |
| CMR + Radiomics | Surface area to volume ratio  Median  Busyness  Large area low gray level emphasis  Gray level non-uniformity  Root mean squared  Large area low gray level emphasis  Mean  Large dependence low gray level emphasis  Sphericity  Contrast  Gray level non-uniformity  Difference entropy  Energy  Sphericity  Joint average  Range  Large area emphasis  Sum entropy | Shape  First-Order  Texture  Texture  Texture  First-Order Texture  First-Order  Texture  Shape  Texture  Texture  Texture  Texture  Shape  Texture  First-Order  Texture  Texture | MYO  MYO  MYO  MYO  MYO  MYO  MYO  MYO  MYO  LV  MYO  MYO  MYO  MYO  MYO  MYO  MYO  MYO  MYO | ED  ES  ES  ES  ES  ES  ED  ES  ED  ED  ED  ES  ED  ES  ED  ES  ED  ED  ES | 0.64 (±0.02)  0.57(±0.06)  0.57 (±0.04)  0.55(±0.04)  0.63 (±0.01)  0.54(±0.05)  0.57 (±0.04)  0.55(±0.06)  0.57 (±0.04)  0.52(±0.05)  0.56 (±0.03)  0.61(±0.01)  0.57 (±0.04)  0.48(±0.03)  0.59 (±0.04)  0.56(±0.05)  0.56 (±0.07)  0.60(±0.01)  0.54(±0.02) |
| CMR + Radiomics + VRF | Surface area to volume ratio  Median  Busyness  Age  Large area high gray level emphasis  Mean  Zone variance  Large area emphasis  Zone variance  Sex  Busyness  Gray level non-uniformity  Root mean squared  Hypertension  Maximum 2D diameter slice  Short run low gray level emphasis  Long run low gray level emphasis  Low gray level emphasis | Shape  First-Order  Texture  Texture  Texture  First-Order  Texture  Texture  Texture  Texture  First-Order  Shape  Texture  Texture  Texture | MYO  MYO  MYO  MYO  MYO  MYO  MYO  MYO  MYO  MYO  MYO  LV  MYO  MYO  MYO | ED  ES  ES  ES  ED  ED  ED  ES  ED  ED  ED  ED  ED  ES  ED | 0.64 (±0.02)  0.57 (±0.06)  0.57 (±0.04)  0.61 (±0.05)  0.55 (±0.04)  0.54 (±0.05)  0.60 (±0.01)  0.60 (±0.01)  0.59 (±0.05)  0.62 (±0.03)  0.57 (±0-04)  0.59 (±0.02)  0.56(±0.05)  0.55(±0.03)  0.55 (±0.04)  0.45(±0.03)  0.46(±0.05)  0.53(±0.03) |

*Abbreviations: CMR, cardiac magnetic resonance imaging; VRF, vascular risk factor, ROI, region of interest, SVM model alone: support vector machine model performance showing the mean and standard deviation using each radiomic feature individually; LV, left-ventricle; RV, right-ventricle; MYO, left ventricle myocardium; ED, end-diastolic, EF, ejection fraction, EDV end-diastolic volume, ESV, end-systolic volume, LV, left ventricle, RV right ventricle, SV stroke volume.*

**Supplementary Table 6: Performance with healthy controls without any cardiovascular disease or stroke**

*.*

|  |  | VRF | CMR | Radiomics | VRF + CMR | VRF + Radiomics | CMR + Radiomics | VRF + CMR + Radiomics |
| --- | --- | --- | --- | --- | --- | --- | --- | --- |
| AF | Accuracy  Sensitivity  Specificity | 0.66(±0.02)  0.65(±0.06)  0.66(±0.05) | 0.65(±0.04)  0.66(±0.05)  0.63(±0.08) | 0.66(±0.07)  0.66(±0.08)  0.65(±0.07) | 0.67(±0.03)  0.65(±0.07)  0.69(±0.04) | 0.72(±0.06)  0.71(±0.04)  0.73(±0.09) | 0.67(±0.07)  0.66(±0.07)  0.67(±0.09) | **0.73**(±0.04)  **0.72**(±0.06)  **0.75**(±0.05) |
|  | AUC | 0.69(±0.04) | 0.68(±0.06) | 0.71(±0.08) | 0.73(±0.04) | **0.77**(±0.06) | 0.71(±0.08) | **0.77**(±0.06) |
| HF | Accuracy  Sensitivity  Specificity | 0.71(±0.04)  0.68(±0.04)  0.74(±0.07) | 0.69(±0.03)  0.64(±0.03)  0.74(±0.08) | 0.68(±0.05)  0.66(±0..07)  0.70(±0.07) | 0.75(±0.04)  0.73(±0.08)  0.77(±0.1) | 0.77(±0.06)  **0.76**(±0.09)  0.77(±0.06) | 0.68(±0.06)  0.66(±0.04)  0.71(±0.09) | **0.78**(±0.05)  **0.76**(±0.08)  **0.79**(±0.05) |
|  | AUC | 0.78(±0.04) | 0.74(±0.03) | 0.77(±0.05) | 0.84(±0.05) | 0.84(±0.06) | 0.76(±0.5) | **0.85**(±0.06) |
| MI | Accuracy  Sensitivity  Specificity | 0.64(±0.03)  **0.65**(±0.03)  0.63(±0.06) | 0.63(±0.03)  0.62(±0.09)  0.63(±0.04) | 0.64(±0.05)  0.64(±0.01)  0.64(±0.05) | 0.64(±0.03)  0.64(±0.09)  0.64(±0.04) | **0.67**(±0.04)  **0.65**(±0.05)  **0.69**(±0.05) | 0.64(±0.05)  0.64(±0.09)  0.64(±0.05) | 0.66(±0.02)  0.63(±0.03)  **0.69**(±0.06) |
|  | AUC | 0.71(±0.03) | 0.68(±0.04) | 0.69(±0.06) | 0.70(±0.04) | **0.74**(±0.03) | 0.68(±0.06) | **0.74**(±0.04) |
| Stroke | Accuracy  Sensitivity  Specificity | 0.62(±0.07)  0.62(±0.08)  0.62(±0.1) | 0.59(±0.04)  0.56(±0.05)  0.61(±0.1) | 0.63(±0.05)  0.60(±0.08)  **0.66**(±0.06) | 0.63(±0.03)  0.60(±0.07)  0.64(±0.08) | 0.63 (±0.03)  **0.64**(±0.07)  0.62(±0.08) | 0.63(±0.03)  0.61(±0.06)  0.65(±0.03) | **0.64**(±0.06)  0.61(±0.08)  **0.66**(±0.06) |
|  | AUC | 0.67(±0.1) | 0.62(±0.03) | 0.68(±0.07) | **0.69**(±0.07) | **0.69**(±0.04) | 0.67(±0.07) | **0.69**(±0.07) |

***Table 6 footnote:***

*Abbreviations: CMR, cardiac magnetic resonance imaging; VRF, vascular risk factor, AF, atrial fibrillation, HF, heart failure; MI, myocardial infarction*
